# Paths to social licence for tracking-data analytics in university research and services

**DOI:** 10.1371/journal.pone.0251964

**Published:** 2021-05-21

**Authors:** Joshua P. White, Simon Dennis, Martin Tomko, Jessica Bell, Stephan Winter

**Affiliations:** 1 Complex Human Data Hub, Melbourne School of Psychological Sciences, The University of Melbourne, Melbourne, Victoria, Australia; 2 Melbourne School of Engineering, The University of Melbourne, Melbourne, Victoria, Australia; 3 Melbourne Law School, The University of Melbourne, Melbourne, Victoria, Australia; University of Pisa, ITALY

## Abstract

While tracking-data analytics can be a goldmine for institutions and companies, the inherent privacy concerns also form a legal, ethical and social minefield. We present a study that seeks to understand the extent and circumstances under which tracking-data analytics is undertaken with social licence—that is, with broad community acceptance beyond formal compliance with legal requirements. Taking a University campus environment as a case, we enquire about the social licence for Wi-Fi-based tracking-data analytics. Staff and student participants answered a questionnaire presenting hypothetical scenarios involving Wi-Fi tracking for university research and services. Our results present a Bayesian logistic mixed-effects regression of acceptability judgements as a function of participant ratings on 11 privacy dimensions. Results show widespread acceptance of tracking-data analytics on campus and suggest that trust, individual benefit, data sensitivity, risk of harm and institutional respect for privacy are the most predictive factors determining this acceptance judgement.

## Introduction

The increased simplicity of acquiring tracking data (spatio-temporal data attached to an identifiable object or person), and their immense utility for logistics, traffic and space management, human resource management, and advertising has turned tracking-data analytics into a revenue goldmine. Yet, the inherent privacy concerns of tracking data turn them equally into a minefield.

One source for tracking data are Wi-Fi networks [1, 2]. Campus-based universities, shopping malls, and hospitals are examples of complex, built environments with varied stakeholders and complex logistical issues that, on one hand, operate such Wi-Fi networks, and on the other need data-driven support for efficient operations. The realisation that tracking data contain information about staff attendance, can support health and safety reporting and compliance monitoring, and can inform space-use optimisation leads to an increased interest on behalf of institutions in active tracking technology deployments [2, 3].

While the operators of the infrastructure will use this data for the optimization of their services, other parties have an interest in this data as well [4]. Other interested parties, such as those whose data is being collected, may consider the use of tracking-data analytics to be invasive, controlling, and only to the benefit of the institution collecting the data, while other possible uses might be accepted by the users as useful, legitimate, or desirable. For example, using tracking-data to monitor staff attendance may be of benefit to an institution, but could largely be to the detriment of workers for whom it could provoke mistrust and threaten their sense of agency and privacy. Clearly, as with any technological advancement, the use of tracking data analytics involves the balancing of various social, ethical, and legal issues. Yet while infrastructure operators often focus only on satisfying the legal requirements that underlie operations, recent scholarship has emphasised the importance of going beyond this minimal threshold to technology deployment, and secure a broader basis for community acceptance.

This notion—termed ‘*social licence to operate*’—while developed primarily in the corporate social responsibility literature to describe the support and acceptance within the community of private companies’ operations, has recently gained traction in the context of large-scale personal data collection and analysis. There are a number of examples of recent controversies in the healthcare sector that exemplify the degree to which a failure to secure social licence for research initiatives can undermine project success; e.g., My Health Record in Australia and Care.data in the UK [5]. Such failures to secure social licence, however, are not limited to the healthcare sector. Indeed, the release of information about the use of Wi-Fi and other big data in learning analytics in Australian universities has been similarly controversial and received backlash from students, regulators and the media [6–9]. Such controversies have also occurred in US universities [10, 11], and may cause negative media attention and attitudes towards institutions, as well as negatively affect the quality of the data collected through reduced compliance or cooperation. These examples illustrate the importance of building social licence and trust amongst the community in a university and education setting if the potential benefits of use of Wi-Fi tracking data are to be realised in the future.

Extensive literature covers the ethically normative requirements for using big data in a variety of contexts [4, 12], including in universities [13]. However, most work in relation to social licence focuses on the legal (i.e., what can entities legally do) and normative frameworks (i.e., what entities should do). However, little work has considered the social licence for big data usage from an empirical or descriptive perspective. While there has recently been an increase in this empirical work, it has generally been limited to the social licence for big data usage in governmental services or the mining industry [12, 14–16]. Here, we complement this gap by focusing on the quantitative analysis of the social licence for data use in research, in the context of data collected and analysed at Universities.

On university campuses, tracking data are often applied to the optimization of campus management, operations, and university services [3]. While, these uses are often legally covered by the Wi-Fi user agreements, a failure to ensure that participants broadly accept their operation can cause negative media attention and attitudes towards the university [6, 9, 10]. Further, these data are also of interest for academic research from a more fundamental perspective of advancing knowledge about the applications and limits of tracking data in a variety of domains. This includes research into privacy protection [17, 18] and obfuscation [19, 20], data cleaning and interpolation [1, 21], pattern detection and prediction, contact tracing [22], and the integration of tracking-data with social media sentiments [23] or with student or staff performance [24]. The use of the Wi-Fi tracking data for research purposes may not be covered in Wi-Fi user agreements, and may require additional informed consent. Wi-Fi users—the whole campus community—may feel uncomfortable with the topics, methodologies, or potential applications of some research and might thus resist having their data contribute towards them. On the other hand, many on campus might benefit from more permissive use of such data through the provision of better university services and management, greater research opportunities for graduate students, and more diffuse public benefits at large.

We thus argue that Universities are a particularly interesting environment to study from the perspective of social licence. The use of Wi-Fi location data in university research and services presents both great opportunities, but also risks such as loss of privacy, community backlash, data breaches etc. This data can be used to greatly benefit the community, by providing the ability to quickly conduct cutting-edge research with real and varied populations, the benefits of which to the community may be immense. In addition to legal requirements, institutional research ethics frameworks also govern Universities, providing extra oversight for data use. There is also a high level of collegiality amongst staff and students. For a productive and flourishing University community, it is crucial that this confidence is not undermined. Trust and confidence are particularly important because in many University institutions, individuals are a ‘captive’ population; individuals need to use the Wi-Fi network to effectively conduct their work and studies. As such, an individual’s ‘freedom’ to opt-out may be fundamentally compromised. For this reason, it is vital to ensure protections are in place for stakeholder privacy, security and risk of harm and it is equally crucial to ensure social licence for trustworthy Wi-Fi location data use before implementation on campus.

We seek to understand to what extent and under what circumstances tracking-data analytics at universities is undertaken under a *social licence*—i.e., with broad community acceptance beyond formal compliance with legal requirements [25]. Understanding the acceptance by Wi-Fi users may help (a) academics to understand sensitivities and limits in advance, (b) ethics boards with formulating policies and guidelines, (c) university governance with a realistic evaluation of user sentiments, and (d) with a broader public debate on acceptable and unacceptable use of tracking data.

To work towards a more nuanced understanding of the parameters of a social licence for the research use of Wi-Fi data on university campuses, in this paper we specifically address two research questions: first, which scenarios for Wi-Fi location tracking do university community stakeholders find acceptable?; and second, what are the factors within those scenarios that can predict this acceptability?

To answer these questions, participants in our study were exposed to a questionnaire presenting hypothetical scenarios depicting the use of Wi-Fi tracking data in research and support services at the University of Melbourne, and rated these scenarios on 11 privacy dimensions before indicating whether each scenario was an “acceptable” use of university tracking data. Using this survey data, we generalise predictive factors of community acceptability for the collection and use of university Wi-Fi location data, and develop a forward-looking predictive model to allow administrators, ethics boards, and other decision-makers to foreshadow the level of university community acceptance for new research and service projects.

## Materials and methods

### Participants

Participants were recruited via online and physical advertisements at the University of Melbourne and were compensated for their participation by being entered into a draw to win $500. To be included in the study, participants were required to be from the University of Melbourne community defined to include students, staff, and any others who regularly attend the University of Melbourne campus. There were no exclusion criteria.

### Materials

Materials were 11 hypothetical scenarios, each of which described a project involving the use of Wi-Fi location data on the University of Melbourne campus. The scenarios were co-developed by a group of key University of Melbourne stakeholders including students, academics, and professional staff to cover a range of plausible university research and service projects that may require the use of tracking-data analytics on campus, and to cover a range of projects with different purposes, benefits, and funding arrangements. All 11 scenarios are reproduced in full at S1 Text, but a summary of each is shown in Table 1. The scenarios all depict uses of tracking-data that are likely to provide benefits to the university community (e.g., students, staff, researchers, people attending campus) or the wider community. This was done to place tracking-data use in a real-world context that requires participants to undergo a ‘privacy calculus’ [26] about whether the benefits of the project outweigh its privacy costs, and give a more ecologically valid understanding of participants attitudes to privacy. By using these real world scenarios with appreciable benefits, we hope to avoid the ‘privacy paradox’ in which people actually give up a lot of their personal data and privacy while still professing strong attitudes favoring personal privacy [27, 28].

**Table 1:** Summary of scenarios presented to participants.

Scenario Name	Scenario Summary
Work Records	University services develop a web-based platform to enable workers on campus to log their working hours and track their working locations through Wi-Fi data, allowing the University to improve service quality and worker safety. Workers usage of the web-based platform is compulsory.
Memory for Where	Researchers collect participants’ location via University Wi-Fi data, and later undertake an experiment in which participants recall where they were at certain times. This research seeks to understand people’s memory for location and determine what factors influence the errors they make. Participants must opt-in to the study and are paid $50 for their data and $15 for completing the memory experiment.
Serving you Better	The University uses Wi-Fi location data and analysis techniques to monitor the use of university food outlets and common areas to better understand the factors that drive the use of these facilities, and how to provide students with a better university experience. No analysis of individual patterns will be undertaken, only aggregated statistical trends. Students can opt-out of the project if they wish.
Safe Campus	Academic researchers collaborate with a start-up firm to use university Wi-Fi data to develop a smartphone app that allows people to be matched with ’walking buddies’ to keep them safe at night on campus.
Student Wellbeing Project	The University uses student data, including their W-Fi location data, in a learning analytics system to help improve mental health and identify at-risk students. Students must opt in to participate, and are paid $20 for doing so.
Project Move	The University collaborates with Yarra Trams to use university Wi-Fi location and timetable data to improve public transport availability and provide information about likely wait-times and occupancy.
Project TRIIBE	University campus shopping and food retailers, collaborating with researchers, use location and internet history data to develop methods to capture and analyse indoor shopping behaviour across shoppers’ physical, online, and social environments, to improve customer experience. Participants are paid $100 for their data and must opt in to participate.
Project QueueSense	Researchers collect Wi-Fi location data at select locations on campus (such as café outlets) to develop algorithms to reduce queueing times at these locations. Coffee will be discounted during the 1-month period of data collection
Project Fluloc	University researchers collect data of social interactions based on indoor Wi-Fi tracking and online health questionnaire data, to assess the role of professional and educational environments in the spread of influenza.
Project Precinct Change Management	University researchers, collaborating with University Services and the Facilities Management Metro, develop algorithms and computational methods to understand the impact of construction disturbances on campus operations and develop methods for improved space use.
Impact of Attendance on Academic Performance	Researchers use Wi-Fi location data to track a cohort of students for one semester to understand how university attendance and presence affects academic and other outcomes.

### Measures

Participants rated these scenarios in relation to 11 privacy dimensions on a 0–5 Likert scale. Table 2 is a list of these dimensions, the question posed to participants in relation to each dimension, and the labels used for its Likert scale. Only the most extreme points of the Likert scale were defined, and a slider bar was used between these points.

**Table 2:** Summary of privacy dimensions on which each scenario was rated.

Dimension	Question	Likert response
Decline Difficulty	How easy is it for people to decline participation in the proposed research?	0 = ‘Extremely easy’
		5 = ‘Extremely difficult’
Private Benefit	How much would private entities benefit from the proposed research?	0 = ‘Not at all’
		5 = ‘Extremely’
Participant Benefit	How much would participants (i.e., the people whose data is being collected) benefit from the proposed research?	0 = ‘Not at all’
		5 = ‘Extremely’
Public Benefit	How much would the public benefit from the proposed research?	0 = ‘Not at all’
		5 = ‘Extremely’
Disproportionality	To what extent are the researchers only collecting the data necessary to achieve the purposes of the proposed research?	0 = ‘Researchers collecting only necessary data’
		5 = ‘Researchers collecting vast amounts of unnecessary data’
Sensitivity	How sensitive is the data to be collected by the proposed research?	0 = ‘Not at all sensitive’
		5 = ‘Very sensitive’
Risk of Harm	How serious is the risk of harm that could arise from the proposed research?	0 = ‘Extremely low risk of harm’
		5 = ‘Extremely high risk of harm’
Trust	How much do you trust the sponsor of the proposed research?	0 = ‘Not at all’
		5 = ‘Extremely’
Data Security	How secure is the data that would be collected from the proposed research?	0 = ‘Not at all secure’
		5 = ‘Extremely secure’
Ongoing Control	To what extent do participants have ongoing control of their data? This includes controlling how and when data is collected, and having access to view and delete data after it is collected.	0 = ‘No control at all’
		5 = ‘Complete Control’
Respect for Privacy	To what extent do you believe the proposed research respects participants’ privacy?	0 = ‘Not at all’
		5 = ‘Extremely’

These 11 privacy dimensions were developed from an extensive literature review and a workshop event in which University of Melbourne stakeholders including students, academics (from Law, Engineering, Computer Science and Psychology) and professional staff from University Services ‘brainstormed’ the relevant privacy and acceptability dimensions that may affect social licence. The literature review considered research that examined the dimensions underlying ethical tracking-data collection and use from both a normative [4, 29, 30] and descriptive/empirical perspective, both qualitative [12] and quantitative [14–16]. The final 11 dimensions were drawn qualitatively from an analysis of these sources. For further details of this process, please see the S1 Text and S5 Table.

### Procedure

Participants completed the following experiment (scripted through Qualtrics) on their own electronic devices through their web browser. Participants were instructed that they were going to be shown 3 scenarios that they should read carefully. These 3 scenarios were randomly drawn from the list of 11 scenarios and shown to them sequentially. For each scenario, participants were given an attention check question which asked, “What was the previous scenario about?”. There were 4 randomly ordered response options to this question: the correct answer for the scenario they had just read, and 3 decoy answers drawn randomly from the correct answers for the other scenarios. Participants that failed at least one of these attention check questions were excluded from analysis.

Participants were then asked to rate each scenario on a 0–5 Likert scale in relation to the 11 privacy dimensions. Participants rated each of their 3 randomly drawn scenarios contemporaneously on each dimension to allow for comparative scaling. While doing so, participants were provided with the scenario text below for them to refer to if necessary. The dimensions were presented to participants in the top–bottom order indicated in Table 2. After rating each scenario on each dimension, participants were asked ‘whether the proposed use of university data in each scenario was acceptable’ and gave a binary yes/no response. Finally, participants were asked some demographic questions: their age, gender, educational attainment, and their relationship to the University of Melbourne.

### Ethics statement

This study received ethics approval from the University of Melbourne’s psychology health and applied sciences human ethics sub-committee, approval number 1955555.1. All participants gave written informed consent prior to participating and were debriefed at the end of the experiment.

### Statistical analysis

#### Descriptive model

Bayesian generalised linear mixed modeling (GLMM) was used to predict participant’s acceptability judgements as an additive function of their privacy dimension judgements. Random intercept effects were included in the model to account for the dependency of the data introduced by different participants rating different scenarios. Thus, all random effects were blocked by participant and scenario. These random effects can be thought of as modelling the acceptability that is inherent in the participant or scenario that is not captured in the model’s fixed parameters (the privacy dimension ratings and interactions therebetween). The privacy dimension Likert ratings were treated as numeric data for the purposes of modeling. The best-fitting model was also estimated with monotonic effects that preserve the ordinal nature of the Likert ratings [31]. This model performed worse for out-of-sample predictive accuracy (see S1 Text), providing support for treating these Likert ratings as numeric in all the analyses reported in this paper.

Posterior distributions of model parameters were estimated using Hamiltonian Markov Chain Monte Carlo No-U-turn Sampling implemented in the R package *brms* [32], a high level interface to Stan [33]. Four chains with 2000 iterations (including 1000 ‘burn-ins’) were used. Uninformative priors were used for the intercept and random effect standard deviation parameters; both were Cauchy distributed with a location parameter of 0 and a scale parameter of 2.5 [34, 35]. Weakly informative priors were used for all fixed effect coefficients: a Laplacian (double exponential) distribution with a location parameter of 0 and a scale parameter of 0.2 (1 / range).

We developed two plausible candidate models: one which included one parameter for each privacy dimension, and one which also included some *a priori* interaction parameters between privacy dimensions (see S1 Table). Model selection between these models was conducted via Pareto-smoothed importance sampling leave-one-out cross validation using the *loo* package [36] in R: the model with the highest expected log predictive density (ELPD) was preferred.

To make inferences about the existence of effects, we used the Region of Practical Equivalence (to a null effect; ROPE) + 89% High Density Interval (HDI) decision rule: infer an effect if the entire 89% HDI falls outside of the ROPE [37, 38]. The region of practical equivalence was deemed to be any effect that causes less than a 5% increase or decrease from the mean acceptability proportion over the entire range of the variable. In this case, the ROPE was thus ± 0.098.

#### Predictive model

Data was randomly split into a training (80%) and testing set (20%) grouped by participant so that no participant’s data was used in both training and testing. 3 models were fit on the training dataset via maximum likelihood estimation and then evaluated on the test dataset, using classification accuracy as the model selection metric. The logistic regression and the mixed effects logistic regression were estimated with *lme4* [39]. ElasticNet logistic regression was conducted using *caret* [40] and *glmnet* [41]. 5-repeat 10-fold cross validation was used to tune regularization hyperparameters, α and λ, via a grid search (all combinations of α and λ for A = {0, 0.05, 0.1, … 1} and Γ = {0, 0.05, 0.1, … 2}), again using classification accuracy as the metric. For prediction in the test dataset, the random intercepts from the mixed effects model were dropped, and only fixed effects were used.

## Results

### Demographics

Participants were 314 members of the University of Melbourne community (198 female, 111 male, 5 prefer not to say, *M*_age_ = 25.63, *SD* = 8.14, range: 17–63 years). All participants gave their informed consent prior to the experiment. Twenty-seven participants failed at least one of three comprehension checks, leaving a final sample of 287 participants (184 female, 98 male, 5 prefer not to say, *M*_age_ = 25.59, *SD* = 8.21, range: 17–63 years).

**Fig 1:**
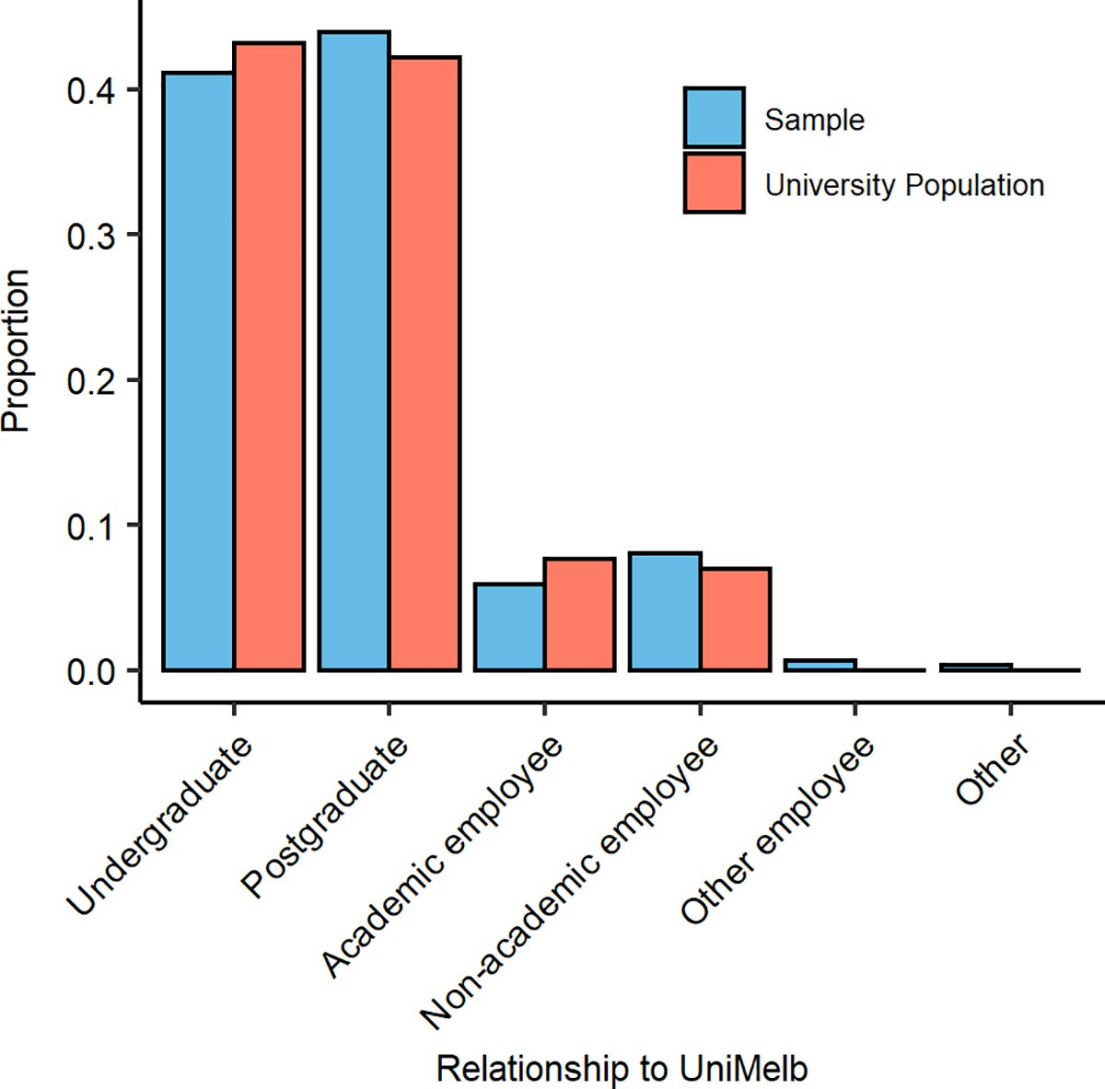
Sample characteristics compared to population. The proportion of participants in our sample (blue) from each ‘relationship to UniMelb’ category compared to that of the university population (red). Note, no population data was obtained for ‘other employee’ or ‘other’ categories.

We endeavoured to match our sample to the distribution of stakeholder types (e.g. undergraduate, postgraduate, academic employee etc.) of the university. Fig 1 shows the proportion of each type juxtaposed with the corresponding population figures taken from the University of Melbourne’s 2019 Annual Report [42]. Our sample is representative of the students and staff of the University of Melbourne, but no population data is available for the number of other employees or others who regularly attend the university campus and our sample likely underrepresented these demographics.

### Acceptance and perception of tracking-data analytics

**Fig 2:**
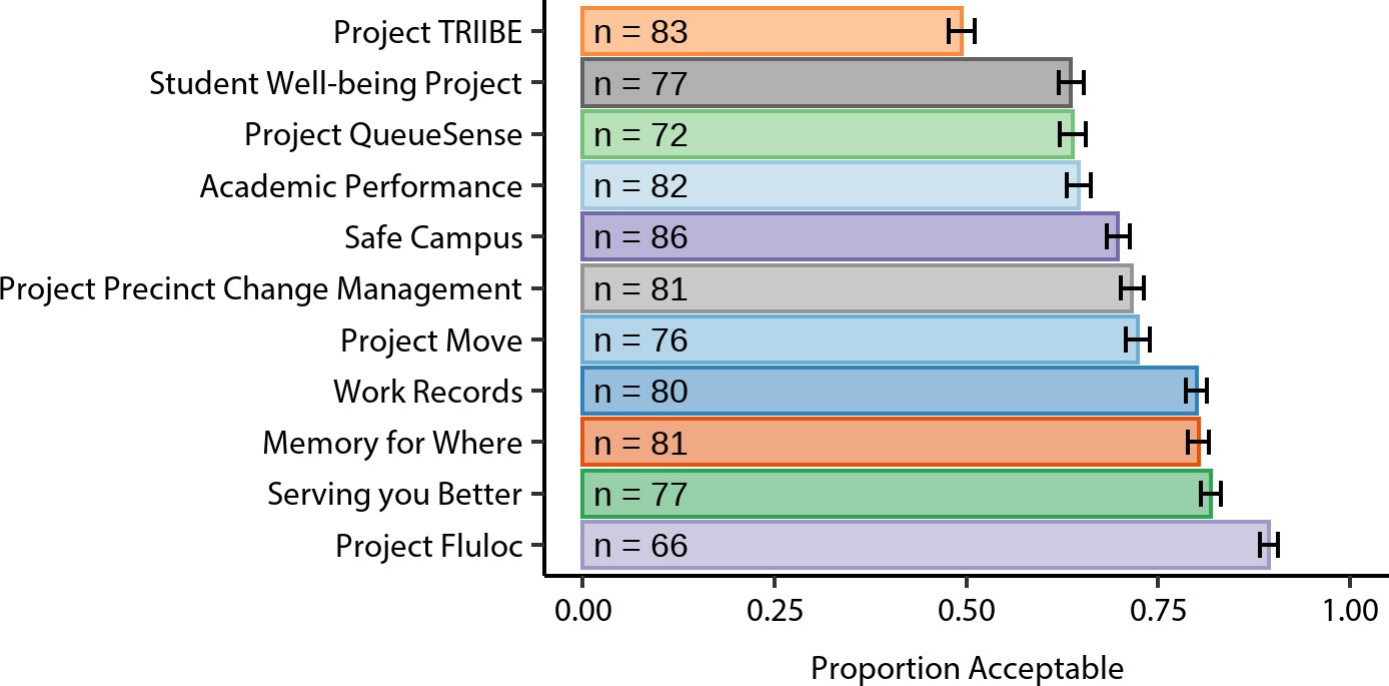
Participant perception of scenario acceptability. The proportion of ‘acceptable’ judgements for each scenario. The sample size for each scenario is listed at the base of the bar chart. Error bars represent standard errors of the mean.

**Fig 3:**
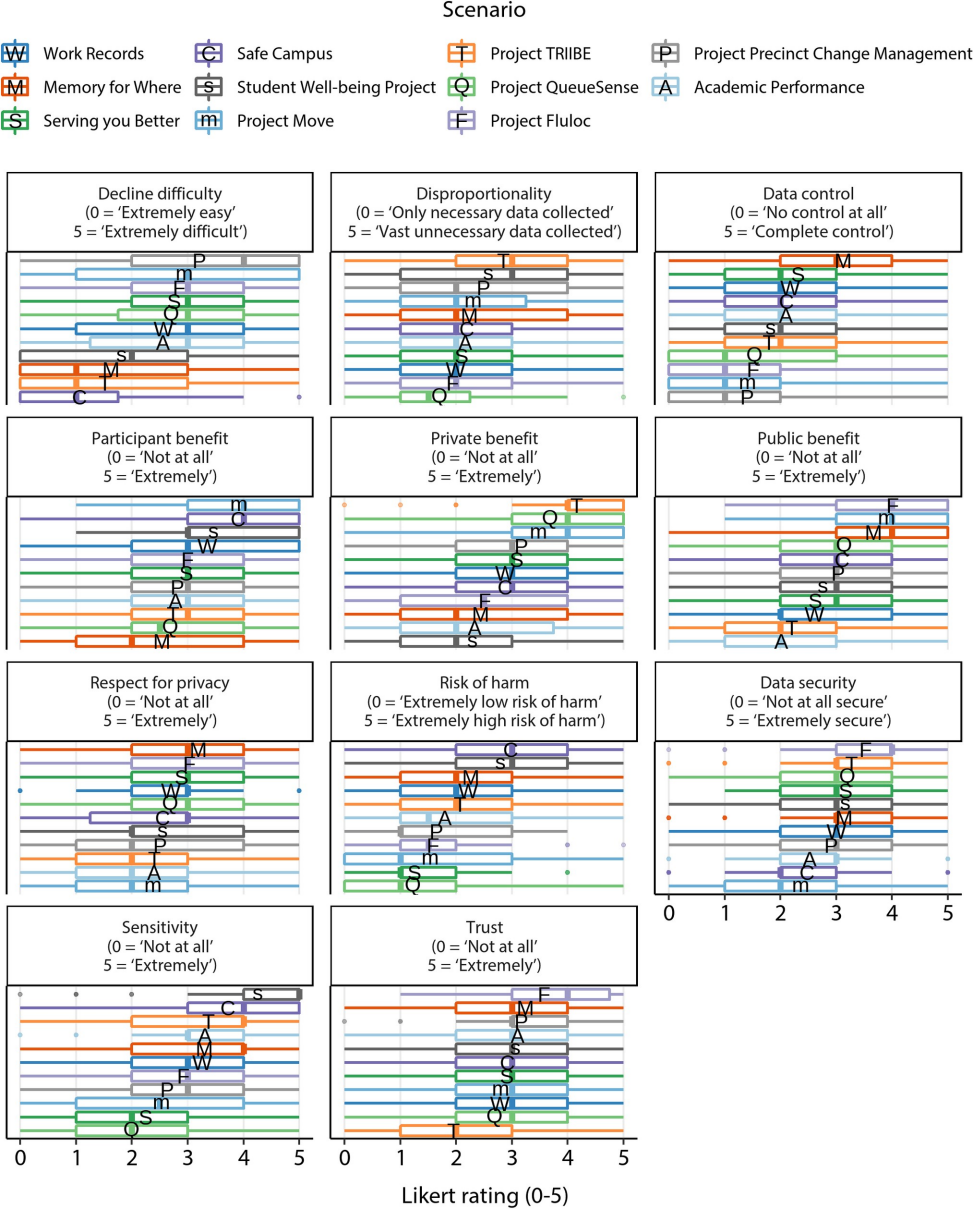
Participant ratings on privacy dimensions, by dimension. Participant ratings of each privacy dimension for each scenario, organised by privacy dimension. Letters on boxplots indicate the mean ratings of each privacy dimension for each scenario.

**Fig 4:**
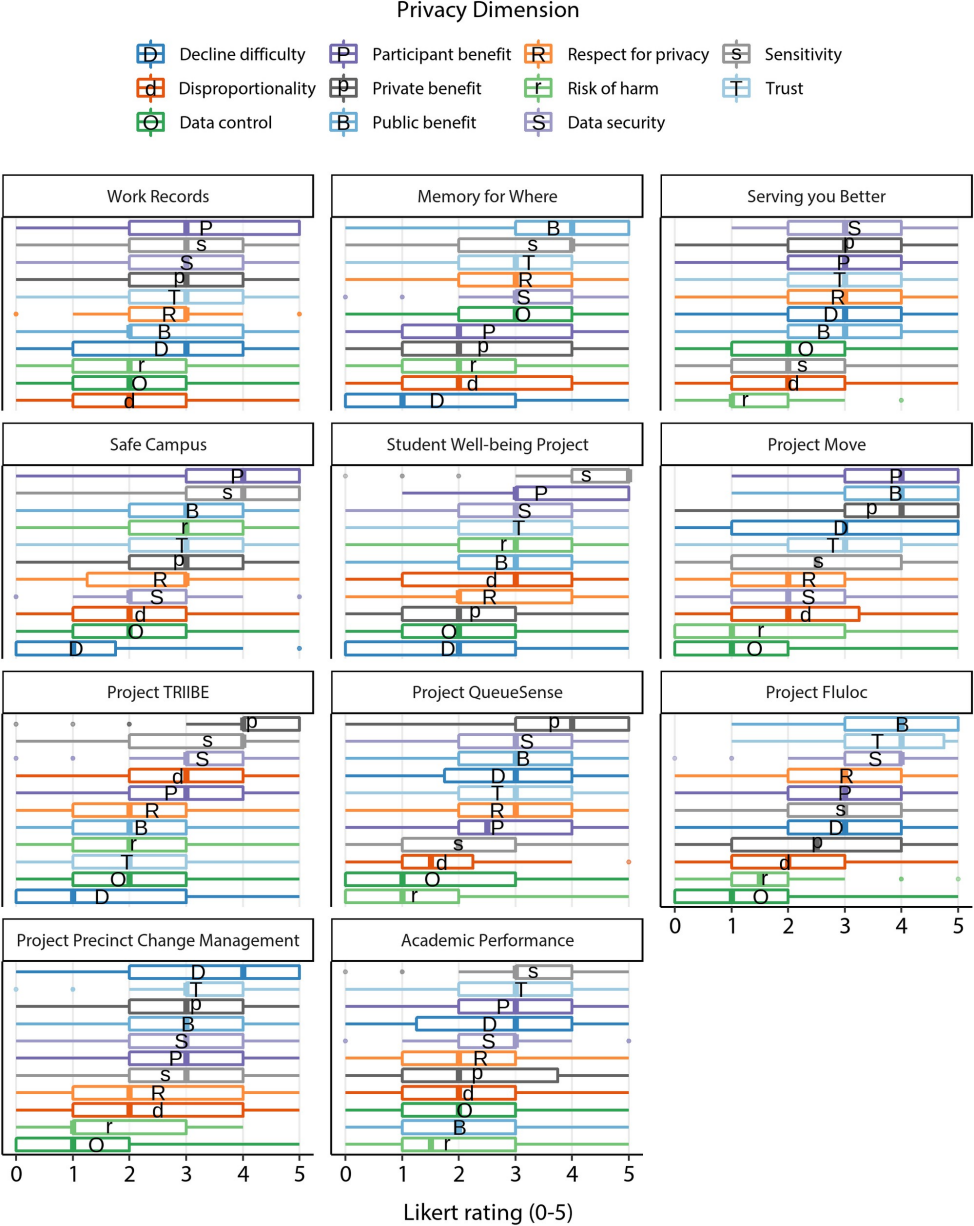
Participant ratings on privacy dimensions, by scenario. Participant ratings of each privacy dimension for each scenario, organised by scenario. Letters on boxplots indicate the mean ratings of each privacy dimension for each scenario.

Fig 2 shows the proportion of participants that labelled each scenario as acceptable. The mean acceptance proportion among the scenarios was 0.715 (*SD* = 0.111). Figs 3 and 4 show participants’ perceptions of each of the scenarios in relation to each of the 11 privacy dimensions (ratings in Fig 3 are ordered by dimension, Fig 4 by scenario). For precise means and standard deviations of these privacy dimension ratings for each scenario see S2 Table.

**Fig 5:**
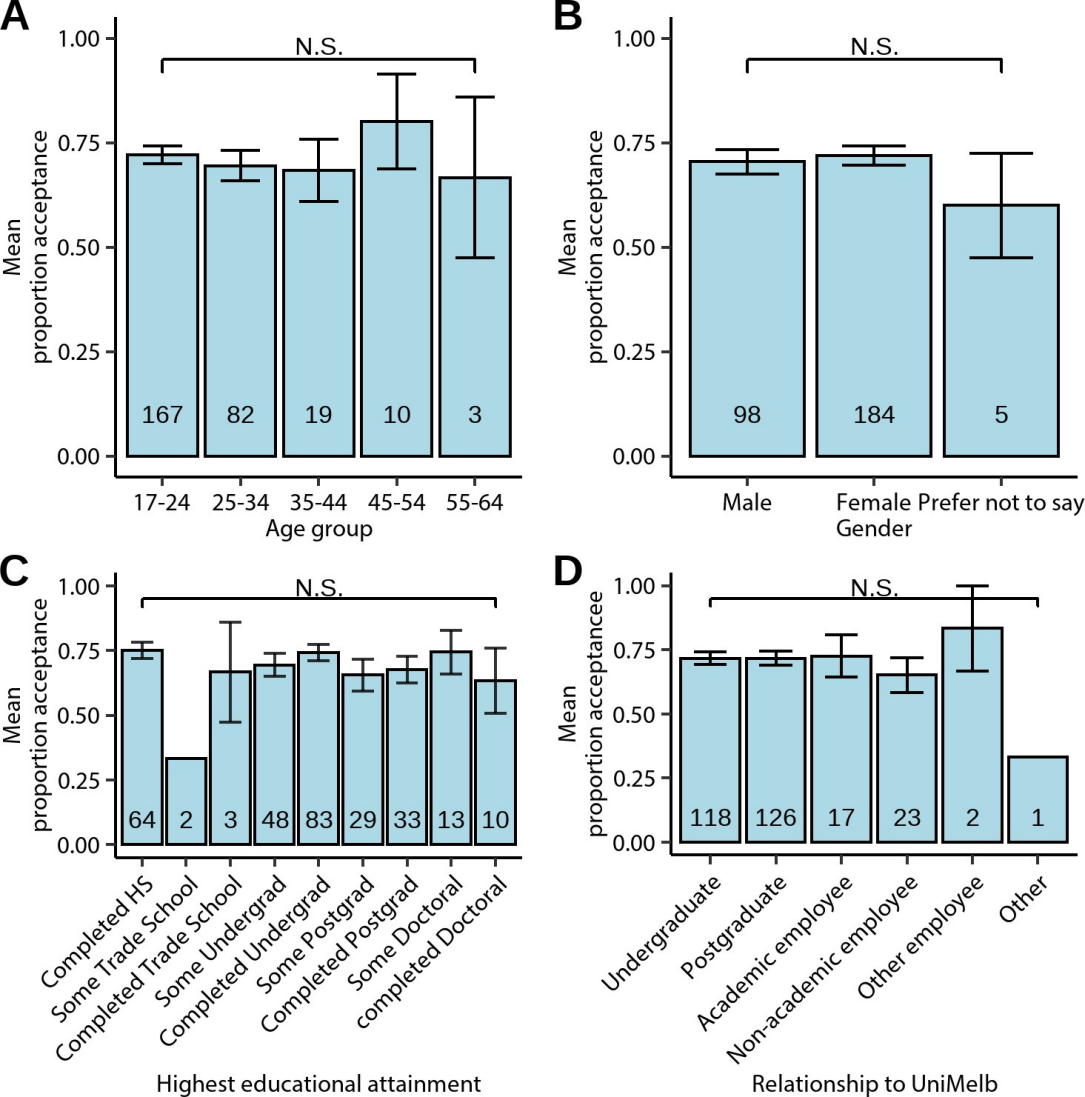
Acceptability of tracking-data analytics by demographics. (**A-D**) Mean participant acceptability proportion broken down by age group (**A**), gender (**B**), Educational attainment (**C**), and relationship to the University of Melbourne (**D**). Acceptability ratings did not differ by any of these demographic variables. Numbers at the bottom of the bars indicate the sample size in that group.

Panels A–D of Fig 5 show mean participant acceptance judgements broken down by demographic variables. Omnibus ANOVA showed that mean acceptance did not differ by gender, *F*(2, 284) = 0.449, *p* = 0.638, relationship to University of Melbourne, *F*(5, 281) = 0.605, *p* = 0.696, educational attainment, *F*(8, 276) = 0.993, *p* = 0.441, or age group, *F*(4, 276) = 0.377, *p* = 0.825. Simple OLS regression analysis also showed that mean acceptance did not differ by age when treated as a continuous variable, *t*(279) = -0.20, *p* = 0.844.

### Predicting acceptance of tracking-data analytics

#### Descriptive model

Bayesian mixed effects logistic regression was used to predict participants’ acceptability judgements as an additive function of their privacy dimension judgements. Two candidate models were estimated: one which included one parameter for each privacy dimension, and one which also included some *a priori* interaction parameters between privacy dimensions (listed in S1 Table).

Model selection was conducted via Pareto-smoothed importance sampling leave-one-out cross validation to maximise the out-of-sample predictive accuracy of the model [36, 43] and the model with the highest expected log predictive density (ELPD) was preferred. On this basis, the model without interactions (ELPD = -345.22, *SE* = 17.54) was preferred to the model with interactions (ELPD = -349.66, *SE* = 18.80). The preferred model had a Nakagawa conditional *R*^2^ of 0.454 (*SE* = 0.034), indicating that 45.6% of the variance in the data was explained by the random and fixed predictor variables, and a Nakagawa marginal *R*^2^ of 0.398 (*SE* = 0.024) indicating that 39.8% of the variance in the data is explained by the fixed predictor variables, i.e., the privacy dimension ratings [44].

**Fig 6:**
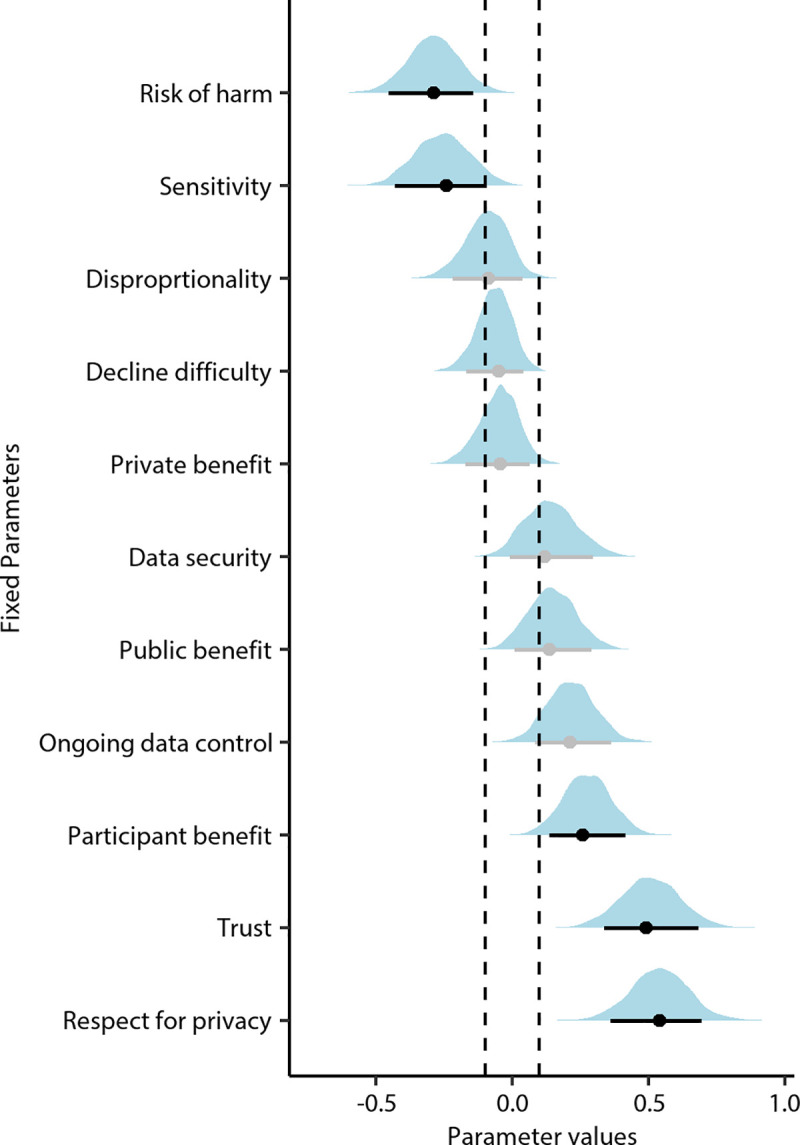
Fixed effect posterior distributions from the preferred model. Point estimates (dots) are posterior modes and intervals are 89% highest density intervals (lines). Parameters for which a practically significant effect was inferred have interval lines coloured black. The region of practical equivalence is shown between the dotted vertical lines. Practically significant effects were inferred when the entire 89% highest density interval fell outside the region of practical equivalence.

Posterior estimates of all fixed model parameters are shown in Fig 6 and listed in S3 Table. Following the Region of Practical Equivalence (to a null effect; ROPE) + 89% High Density Interval (HDI) decision rule, we inferred a practically relevant effect when the entire 89% HDI falls outside of the ROPE [37, 38]. On this basis, we inferred a predictive effect on acceptability judgements for ratings of respect for privacy, trust in the research sponsor, risk of harm, participant benefit from the tracking-data analytics, and data sensitivity. The belief that the research respected people’s privacy had the biggest predictive effect on acceptability judgements, with a 1-unit increase in this rating resulting in a 1.72-factor increase in the odds of judging the project as acceptable. On the other hand, the smallest practically relevant effect was for the sensitivity of the data, with a 1-unit increase in this rating resulting in a 1.29-factor decrease (i.e., a 0.78-factor increase) in the odds of judging the project as acceptable. Finally, the posterior distributions of the random intercept effects are shown in S1 Fig.

#### Predictive model

We also developed a predictive model to assist decision-makers to estimate the social licence of future university research and service projects. We trained 3 models on a training dataset: a logistic regression, a mixed effects logistic regression with random intercepts for scenario and participant, and an ElasticNet logistic regression with regularisation hyperparameters, λ and α, chosen via 10-fold cross validation. The mixed effects logistic regression model had the best prediction accuracy (84.31%) on the test data set when compared to the logistic regression model (83.66%) and the ElasticNet logistic regression model (83.66%). The regularisation hyperparameters of the ElasicNet regression were λ = 0 and α = 0.15.

The preferred model—the mixed effects logistic regression—had a Cohen’s Kappa of 0.61, indicating that it had ‘good’ or ‘substantial’ classification performance [45, 46]. S3 Table shows the parameter coefficients for this model. Given privacy dimension ratings for a new project, these coefficients can be used to predict the likelihood of future acceptability judgements (via the logistic function). To facilitate the ease of this process, we have developed a simple online app (available at https://whitejp.shinyapps.io/lumas-predictive-model/) [47] in which, after entering ratings on each dimension, a user is given a probability in return. This probability can model the percentage of the university community that would accept a project with those ratings.

## Discussion

In this study we aimed to explore and understand the social licence, or lack thereof, for the use of Wi-Fi location data in university research and service provision. More specifically, we sought to understand whether, to what extent, and under what circumstances, there is social license for location data collection and use on university campuses. To answer these questions, we presented participants—members of the University of Melbourne community (students, staff, and others)—with 11 hypothetical scenarios depicting the use of Wi-Fi location data on campus. We asked them to rate these scenarios in relation to 11 privacy dimensions, and finally to indicate whether they believed that the use of data in the scenario was acceptable. We then used participants’ ratings on these 11 privacy dimensions to predict participant attitudes of acceptability, hoping to understand which dimensions are most predictive of social licence.

### Understanding the social licence for tracking-data analytics

Our data generally support the notion that, for the hypothetical projects used in this study, there is wide support for the use of university Wi-Fi location data in university research and services. Indeed, the mean acceptance proportion of the scenarios was 71.5%. and all but one (Project TRIIBE) gained more than 50% acceptance. However, social licence is not conceptualised as merely a majoritarian enterprise but rather as requiring the broad support of the community [48]. Yet, even incorporating a higher acceptance criterion, our data is promising: four of the scenarios (Work Records, Memory for Where, Serving you Better, and Project Fluloc) exceeded 80% acceptability. Of course, exactly where the acceptability criterion should be set by decision-makers as the minimum to establish social licence is likely to depend on contextual factors, and is beyond the scope of this research.

Another consideration relevant to understanding social licence is not just the degree of acceptance of a project, but also how this acceptance is distributed; that is, support for a project must be manifest across the various constituent groups of a community [49]. Here, our results are promising: acceptability judgements did not differ as a function of gender, age, or educational attainment, suggesting that the acceptance of tracking-data analytics is not just concentrated to a few groups. Most relevantly, there were also no differences in acceptance judgements between staff (both academic and non-academic) and students (both under- and post-graduate). Given the different interests and incentives between these groups, it was perhaps surprising that acceptance ratings did not differ [48].

### Factors predicting the acceptability of tracking-data analytics

Our results also give insight into the factors that are most predictive of the acceptable uses of tracking-data analytics on the University of Melbourne campus. Here, we find evidence that the most predictive factors are the perception that the research team respects individual privacy, the trust in the research team, the degree to which people personally benefit from the project, the sensitivity of the data being collected and used, and the risk of harm inherent in the project.

Surprisingly, however, the perception of the public benefit of a project did not show strong evidence of increasing the likelihood of project acceptability, nor did the perception of private or commercial benefit; only a personal benefit did. Thus, clearly identifiable and more imminent benefits that flow directly to those whose data are being collected and used, are more likely to increase social licence than more diffuse, amorphous public benefits.

Our findings also suggest limited predictive ability of a one-time opt-in/opt-out distinction on acceptability. Indeed, we found that perceptions of the initial difficulty of declining to have one’s data collected and used in a project was less strongly predictive of acceptance than perceptions of how much ongoing control over their data users had (e.g., control over how and when one’s data was collected, and personal ability to view and delete their data). This may have implications for privacy law which, in Australia and often elsewhere, considers consent as a requirement, not ongoing control of data [50].

Finally, the strong effect of trust in increasing the likelihood of acceptance in our sample is in line with previous work which suggested that trust was the primary factor underlying social licence in a very different context—for a proposed coal seam gas mine [51]. Similarly, the strong effect for ‘respect for privacy’ shows how acceptance of privacy-encroaching technologies relates to peoples’ perceptions of how such technologies affect their privacy. As such, our findings are broadly in line with the notion that people undertake a privacy calculus in which they weigh the benefits of a technology with the negatives and risks of their privacy being compromised [52].

### Estimating the acceptability of future tracking-data analytics projects

We further provide a simple approach to estimate the social licence of future university research and service projects that may be of use to university ethics boards or decision-makers. We have built a forward-looking predictive regression model which takes ratings of a university project that involves location data collection and analytics on 11 dimensions and predicts what proportion of the university community, given those ratings, will consider the project acceptable. Decision-makers could use this to estimate the acceptability for their proposed projects. To best do so, decision-makers could get a small sample of independent ratings for their project on the 11 dimensions, take the mean rating for each dimension, and input these into the model to obtain an estimate of the proportion of university community members who would likely view the scenario as acceptable, given the scenario ratings. To facilitate this, we have created an online app (available at https://whitejp.shinyapps.io/lumas-predictive-model/) [47]. This ability to prospectively estimate acceptability will allow decision-makers to either drop projects with little prospect of community support, or to make changes to project details (that are reflected by altered independent ratings on the 11 dimensions) to increase community acceptability.

### Limitations

We should be careful not to extend these findings too far beyond the university domain. In particular, we should be careful not to generalise to significantly different contexts such as government surveillance for which there are specific considerations and a large literature [53–56]. Future work should consider the social licence for tracking-data use and collection in a wider context and sample [57–59]. Crucially, social licence requires openness and transparency to inspire trust and confidence in uses of data. The authors’ note with emphasis that social licence is highly context dependent, and hope that the innovative methodological process described in this paper can be used to help uncover trustworthy uses of data in a given context, as part of an open dialogue with those who provide the data in the first instance. While the methodology offers a process for predicting acceptable uses, it is not a panacea but an additional tool in the suite of governance mechanisms for trustworthy data use.

### Conclusion

These findings show that a large majority of the University of Melbourne community find the hypothetical scenarios presented to them acceptable. Further, our research suggests that trust of the research sponsor, the belief that they respect people’s privacy, the benefit to participants of the research or service, and the sensitivity of the data collected, and the risk of harm imposed by the research, are all important factors which help determine the social licence of tracking-data analytics on university campuses. Researchers, university management, and those collecting location and other sensitive data, could use these results to cater their data collection and analytics methodology to community commands and expectations. Indeed, doing so will generally increase the efficacy of the data collection and avoid pushback, protest and negative publicity [60] that may arise from inadvertently stepping out of their social licence.

Considerations of the parameters of social licence of tracking-data analytics are only likely to grow in coming years as tracking-data collection and analysis methodologies become easier and more pervasive, and as a result of the widespread uptake of privacy-encroaching tracking technologies by governments around the world in response to the COVID-19 pandemic [57–59, 61].

## Supporting information

S1 Text Supplementary materials and methods, and results. (DOCX)

S1 Fig Random effect posterior distributions. **(A)** Posterior distributions of scenario random intercepts from the preferred model. Point estimates are posterior modes and intervals are 89% highest density intervals. **(B)** Summary of participant random intercept posterior distributions. Black point estimates are posterior means, red point estimates are posterior modes, and blue intervals are 89% highest density intervals.
(TIF)

S1 Table Interaction effects included in candidate model. (DOCX)

S2 Table Mean and standard deviations (SD) of participant ratings on privacy dimensions for each scenario. (DOCX)

S3 Table Parameter posterior distribution summary statistics for model parameters. (DOCX)

S4 Table Parameter coefficients for predictive model. (DOCX)

S5 Table Developing final privacy dimensions from workshop dimensions. (DOCX)
