# Supplementary material for: Paths to social licence for tracking-data analytics in university research and services
Source: PLoS One. 2021 May 21;16(5):e0251964. doi: 10.1371/journal.pone.0251964 (PMC8139460; doi:10.1371/journal.pone.0251964)
Supplement: S1 Text — (DOCX) [file pone.0251964.s001.docx]

S1 Text. Supplementary Materials and Methods, and Results.

**Materials and Methods**

***Scenarios***

Below we reproduce the final scenarios in full as presented to participants.

Project Title: **Work Records**

Workers from overseas in temporary contracts often face dangerous employment conditions in which they are often underpaid or even harassed when working night shifts in deserted buildings. In this project, researchers will develop a web-based platform that enables foreign temporary workers on campus to log their working hours and track their working locations, and allows University Services to access this data to improve service quality and worker safety. Researchers will be allowed to access the data for advanced data analytics, optimization of tours, and prediction of work behaviour. The research results will be published in scientific journals and conferences, with a focus on the algorithms developed and the experimental validation of them. All published data and diagrams will be aggregate results. The original data will be stored in a secured database for five years and then destroyed, as per university data retention policy. The novel methods developed can then be deployed in facility management services in the future.

Project Title: **Memory for Where**

Project Innocence estimates that between two and five percent of people in prison have been wrongfully convicted. Juries will often attribute the provision of a false alibi to guilt; however, it is not uncommon for people to misremember where they were. In this study, researchers will investigate people’s ability to recall where they were at a given time to determine what factors influence the errors they make. The ultimate objective is to provide the legal system with a better understanding of when a faulty alibi is likely to occur and to prevent false convictions. ​Researchers will collect location data via Wi-Fi connections when participants log onto a university router. Subsequently, participants will also be asked a series of questions about where they were at certain times. Only results aggregated over many people will be released. These will appear in academic papers. All participant data will be stored in a secured server hosted by the University of Melbourne. All transmission of information across networks will occur in encrypted form. Participants will have the capacity to delete their data. Funds for the project come from the Australian Research Council Discovery Program DP312859467. Participants must opt in and will be paid $50 for their data and $15 for completing the memory experiment.

Project Title: **Serving You Better**

As part of its redevelopment of the campus, the University would like to introduce monitoring of the use of food outlets and common areas in order to understand better the factors that drive use of these facilities and how we can provide students with a better experience. A combination of technologies will be used, including thermal imaging in indoor areas to detect the presence of people and collection of data from Wi-Fi access points showing the number of devices connected and the duration of stay in an area. The data from the Wi-Fi access points will have login and device IDs removed and replaced with randomly generated IDs. This data will be analysed for statistical purposes only. No analysis of individual behaviour patterns will be undertaken. The data will be securely housed on AWS servers and analysed by University staff in conjunction with other data, such as weather and timetable data. The results will be used to inform University campus strategy, for example by identifying factors that influence the use of indoor and outdoor spaces in order to better meet future demand. The data will be made available to researchers on a case-by-case basis following ethics clearance from the University. Aggregate (i.e., not individual) data showing trends will be published as open data for reuse by the broader community. People who do not wish for their data to be stored can email the researchers for it to be deleted.

Project Title: **Safe Campus**

In the era of #metoo, there is growing awareness that the campus is not necessarily a safe environment after dark. Academic researchers will engage with a start-up firm to develop a walking companion by night app. The project will be funded by the Australian Research Council through their Linkage Program. Registered users of this app will consent to be tracked continuously and to receive messages in set time windows about possible companions nearby for a real-time matching process. They also accept that their identity and location will be revealed to a matching companion, similar to a taxi service. Twelve hours after a matched walk, the record of the identities will be deleted. The tracking data, however, will further be used for data analytics on pedestrian footfall on campus in order to make travel recommendations. The academics plan to publish their data analytics solutions and the start-up will offer an innovative service to people staying on campus.

Project Title: **Student Well-Being Project**

Universities are facing rising mental health problems amongst students, raising questions about whether universities are providing enough support for student emotional and mental health. As a result, the University of Melbourne is investing in a learning analytics system that will pull together different kinds of data to identify at-risk students. The system combines individual level data, such as attendance and assessment data, with aggregated (i.e., not individual) data such as location data via Wi-Fi connections when people log onto a university router. The system will generate ‘flags’ that are associated with events and movements that are potential signs of concern. Multiple flags will trigger an early warning alert and an intervention measure such as a meeting with a student support professional. In the first stage of the project, University of Melbourne researchers will trial and perfect the system ready for roll out next year. Researchers will access participants’ personal data including attendance and assessment results, and track participant location (via their Wi-Fi connections) when they are on campus. No personal information will be released, but researchers will publish the study’s findings, including a prototype system, in academic papers. All data will be stored in a secured server hosted by the University of Melbourne for 3 years, before being destroyed. Funds for the project come from the University of Melbourne, and participants will be paid $20 for their participation in their study, for which they must opt in.

Project Title: **Move**

At particular peak times, the tram stops around campus are overcrowded. Researchers suggest improving the situation by short-term predictions of public transport demand from university timetable information, theatre occupancy data collected from the Wi-Fi network, weather data, and machine learning of correlations (patterns) over time. For this purpose, they are requesting access to location data of the population (taken via participants’ Wi-Fi connections) on campus over the past year and in real-time. Yarra Trams agrees to sponsor the research project, expecting to adapt their frequencies more flexibly to the actual demand. People, equipped with information about demand and remaining waiting times, might choose alternative transport stops, alternative modes of transport, or just decide to have a coffee first.

Project Title: **Project TRIIBE**

University campus shopping and food retailers currently do not have detailed information about their customers, their shopping patterns and information needs. This puts them at a disadvantage compared to online retailers who have detailed user statistics. Project TRIIBE researches methods to capture and analyse indoor shopping behaviour across shoppers physical, online and social environments, to improve customer experience. The project will collect Wi-Fi connection data and internet access history per visitor. ​ ​The research team will develop privacy-aware recommendation methods based on aggregate online and physical behaviour. The project will release only a technical description of the machine learning model and a method for how a recommender system implementing this model can be developed, as well as the estimate of the relative significance of physical, online and social behaviours for recommendations. Only aggregate (i.e., not individual) statistical information will be released in academic papers. Users’ data will be stored in an encrypted format, on a secure server and accessible only to the university research team sponsored by ARCLP792534861. Participants will be paid $100 for their data for a month, and must opt in to participate. Industry partners will use only the developed method to improve their services, with their own data.

Project Title: **Project QueueSense: Measuring Queuing Behaviour**

The need to optimise the workforce and quality of service in retail, airports and front-desk services requires an efficient means to monitor queue length and service times. This project will develop methods to measure queue times based on Wi-Fi signal sensing. A few selected locations (such as café outlets) around the University will be re-configured and equipped with Wi-Fi sensing devices to collect data about customers present on premises, and the length of time spent in queues. The data collection will run over a one-month period. Only information about Wi-Fi equipped devices will be collected. Shoppers will be notified that their dwell time will be monitored while they purchase refreshments on premises through posters placed outside the cafes. Coffee will be discounted during the period of data collection. Only aggregate (i.e., not individual) statistical information will be released in academic papers. Users’ data will be stored in an encrypted format, on a secure server and accessible only to the university research team sponsored by ARCLP849537612. The project is conducted with Ethics approval from the University of Melbourne. Industry partners will use only the developed method to improve their services, with their own data.

Project Title: **Project Fluloc: Understanding the Spread of Influenza**

Epidemiological models of influenza spread have been previously developed to predict seasonal influenza epidemics in entire nations, and more recently, in large cities. It is well documented that co-location at work and at schools contributes to the spread of the flu. This project will collect data of social interactions based on indoor Wi-Fi tracking across campus to assess the role of professional and educational environments in the spread of influenza. The project will collect Wi-Fi connection data per user, complemented with an online health status questionnaire to all users of the university Wi-Fi, during the flu period of 2020. The research team aim to improve epidemiological modeling methods for urban precincts. The project will release, in academic papers, a technical description of the model and aggregate-level (i.e., not individual) statistics about the influenza season in 2020. The model will inform epidemiological management practices across educational and professional environments. Data will be stored in an encrypted format on a secure server accessible only to the university research team. The project is sponsored by the Department of Health and has Ethics approval from the University of Melbourne.

Project Title: **Project Precinct Change Management**

Large university campuses embedded in the urban environment are regularly impacted by large infrastructure works, such as the Metro tunnel. The ability to analyse the population dynamics on campus in a fine-grained manner would allow the university to optimise campus disturbances by reallocating space-use and making time-management and scheduling modifications for university activities. In this project, researchers will collect individual data from Wi-Fi users on campus and aggregate it to explore statistical data analytic methods enabling researchers to quantify the impact and economic cost of construction disturbances on campus operations, and research statistical optimisation methods for improved space use. This is an applied research project supported by the University Services and Facilities Management Unit. Findings of the project will directly inform operations on campus during the Metro tunnel construction and may inform further management guidelines. The research will be conducted with ethics approval from the University of Melbourne. No individual data will be released beyond a highly securitised server at the University of Melbourne. Aggregate data analytics will be produced in a dashboard environment for University Services and used as illustrations in publications and reports. Only aggregate-level (i.e., not individual) data will be used in publications.

Project Title: **The Impact of Attendance on Academic Performance**

Attendance in lectures is falling. As students juggle competing family and work commitments, the number of students appearing in class can drop 60-70% during the period of a semester. While some lecturers are concerned that the quality of the educational outcomes that universities provide is compromised by this dynamic, others contend that online materials provide a superior student experience, especially in large classes. In this study, researchers will track a random cohort of students for one semester and analyse how much time they are spending on campus and which classes they are attending. Their location data will be cross referenced with their academic results at the end of the semester. In order to minimize the impact of selection effects, researchers will use previous academic performance to account for variation in ability between participants. Students must opt-out if they do not wish their data to be recorded. The results will be used to guide university decision-making about the relative emphasis that should be given to online and offline activities. Only aggregate (i.e., not individual) results will be published.

***Developing the Privacy Dimensions***

Participants at the LUMAS workshop were placed into small groups and performed a best-worst ranking of 6 randomly drawn (of the 12) draft scenarios. Afterwards, participants discussed the various dimensions that influenced their acceptability in terms of best to worse scale and a preliminary list of relevant dimensions was made. The 12 draft scenarios were then analysed in terms of these dimensions in small groups. Afterwards, the whole group identified dimensions that they felt were relevant but were not raised by their scenarios, as well as dimensions raised by their scenarios but had not yet been captured, as well as dimensions they felt were missing.

This list of dimensions was then reduced to the final l1 dimensions to make the experiment shorter for participants and thus maximise the quality of collected data. This was done by a qualitative process in which the original dimensions were combined to higher-order or similar categories, removed when not relevant to the scenarios or too difficult to experimentally manipulate in the present experiment. This process was also informed by prior empirical and normative work which considered the dimensions or factors underlying the acceptability of tracking and surveillance (cited in the main paper). S4 Table is a summary of how the 24 workshop dimensions were reduced to the 11 final dimensions.

**Results**

To assess the reasonableness of our assumption to treat the ordinal Likert data as numeric in our modeling, we compared the best-fitting model presented in the paper (i.e., with no interaction effects) with the same model except treating predictor variables as ordinal, and parameterizing them with monotonic effects. The model using monotonic effects (ELPD = -348.35, *SE* = 18.63) fit worse than the best-fitting model presented in the paper (ELPD = -345.22, *SE* = 17.54). For further details of this analysis, see <https://osf.io/mx948/>.
