# Supplementary figures and images for: Paths to social licence for tracking-data analytics in university research and services
Source: PLoS One. 2021 May 21;16(5):e0251964. doi: 10.1371/journal.pone.0251964 (PMC8139460; doi:10.1371/journal.pone.0251964)

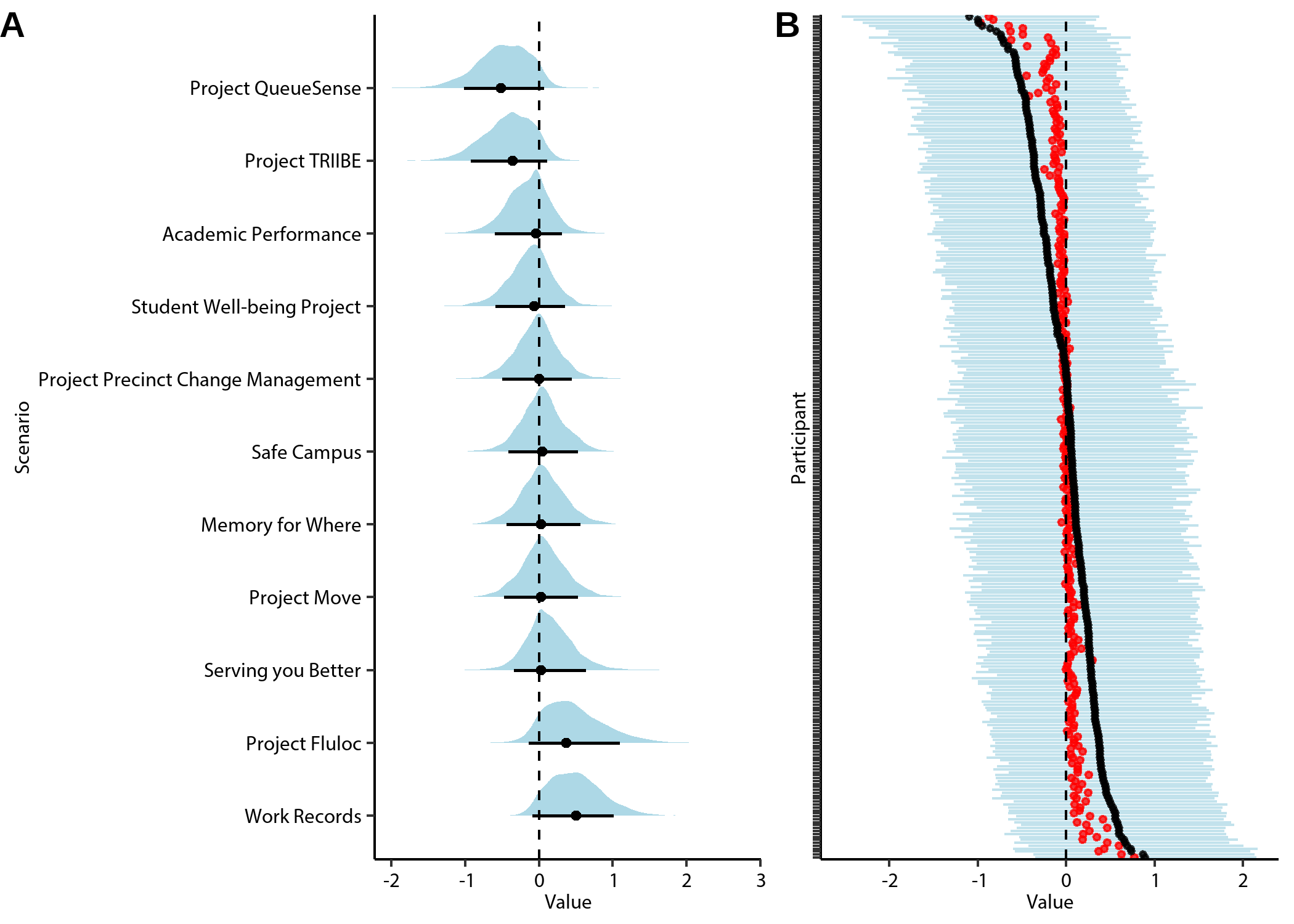

Supplement: S1 Fig — (A) Posterior distributions of scenario random intercepts from the preferred model. Point estimates are posterior modes and intervals are 89% highest density intervals. (B) Summary of participant random intercept posterior distributions. Black point estimates are posterior means, red point estimates are posterior modes, and blue intervals are 89% highest density intervals. (TIF) [file pone.0251964.s002.tif]
