# Supplementary material for: Paths to social licence for tracking-data analytics in university research and services
Source: PLoS One. 2021 May 21;16(5):e0251964. doi: 10.1371/journal.pone.0251964 (PMC8139460; doi:10.1371/journal.pone.0251964)
Supplement: S1 Table — (DOCX) [file pone.0251964.s003.docx]

**S1 Table.** **Interaction effects included in candidate model.**

| A Priori Interaction Effects | |
| --- | --- |
| Private benefit, Participant benefit, & Public benefit | Private benefit & Participant benefit |
| Private benefit & Public benefit | Participant benefit & Public benefit |
| Risk of harm & Decline difficulty | Risk of harm & Ongoing control |
| Security & Sensitivity | Sensitivity & Ongoing control |
| Trust & Sensitivity | Trust & Risk of harm |
| Trust & Security |  |
