# Supplementary material for: Paths to social licence for tracking-data analytics in university research and services
Source: PLoS One. 2021 May 21;16(5):e0251964. doi: 10.1371/journal.pone.0251964 (PMC8139460; doi:10.1371/journal.pone.0251964)
Supplement: S2 Table — (DOCX) [file pone.0251964.s004.docx]

**S2 Table. Mean and standard deviations (SD) of participant ratings on privacy dimensions for each scenario.**

| **Scenario** | **Privacy Dimension** | | | | | | | | | | |
| --- | --- | --- | --- | --- | --- | --- | --- | --- | --- | --- | --- |
|  | **Decline difficulty** | **Private benefit** | **Participant benefit** | **Public benefit** | **Disproportionality** | **Sensitivity** | **Risk of harm** | **Trust** | **Data security** | **Ongoing data control** | **Respect for privacy** |
| Work Records | 2.56 (1.59) | 2.88 (1.47) | 3.35 (1.39) | 2.61 (1.41) | 1.99 (1.27) | 3.26 (1.35) | 2.21 (1.37) | 2.79 (1.32) | 3.00 (1.35) | 2.17 (1.42) | 2.70 (1.26) |
| Memory for Where | 1.62 (1.53) | 2.43 (1.64) | 2.53 (1.47) | 3.67 (1.21) | 2.23 (1.65) | 3.30 (1.42) | 2.26 (1.42) | 3.23 (1.29) | 3.14 (1.17) | 3.12 (1.44) | 3.19 (1.25) |
| Serving you Better | 2.75 (1.42) | 3.08 (1.37) | 2.97 (1.50) | 2.62 (1.36) | 2.09 (1.50) | 2.25 (1.34) | 1.25 (1.03) | 2.91 (1.16) | 3.17 (1.11) | 2.31 (1.35) | 2.88 (1.40) |
| Safe Campus | 1.06 (1.30) | 2.87 (1.63) | 3.91 (1.10) | 3.10 (1.32) | 2.19 (1.38) | 3.72 (1.23) | 2.98 (1.54) | 2.92 (1.31) | 2.48 (1.35) | 2.10 (1.46) | 2.55 (1.32) |
| Student Well-being Project | 1.81 (1.51) | 2.29 (1.50) | 3.45 (1.23) | 2.75 (1.48) | 2.57 (1.58) | 4.25 (1.14) | 2.79 (1.45) | 3.05 (1.35) | 3.16 (1.27) | 1.82 (1.55) | 2.55 (1.46) |
| Project Move | 2.93 (1.77) | 3.47 (1.35) | 3.91 (0.97) | 3.89 (1.11) | 2.30 (1.55) | 2.53 (1.46) | 1.53 (1.31) | 2.79 (1.29) | 2.36 (1.45) | 1.41 (1.31) | 2.37 (1.54) |
| Project TRIIBE | 1.52 (1.66) | 4.16 (0.99) | 2.73 (1.34) | 2.20 (1.39) | 2.84 (1.36) | 3.37 (1.30) | 2.07 (1.45) | 1.95 (1.34) | 3.28 (1.25) | 1.80 (1.40) | 2.40 (1.40) |
| Project QueueSense | 2.69 (1.48) | 3.68 (1.15) | 2.68 (1.41) | 3.14 (1.40) | 1.69 (1.27) | 1.99 (1.38) | 1.22 (1.28) | 2.68 (1.27) | 3.19 (1.13) | 1.53 (1.36) | 2.68 (1.41) |
| Project Fluloc | 2.85 (1.56) | 2.52 (1.51) | 3.00 (1.43) | 4.02 (0.97) | 1.94 (1.47) | 2.92 (1.29) | 1.59 (1.21) | 3.58 (1.24) | 3.53 (1.19) | 1.52 (1.36) | 3.03 (1.41) |
| Project Precinct Change Management | 3.21 (1.57) | 3.17 (1.38) | 2.81 (1.31) | 3.04 (1.31) | 2.49 (1.38) | 2.63 (1.37) | 1.64 (1.19) | 3.17 (1.24) | 2.91 (1.30) | 1.41 (1.30) | 2.51 (1.51) |
| Impact of Attendance on Academic Performance | 2.55 (1.49) | 2.33 (1.62) | 2.78 (1.46) | 2.01 (1.35) | 2.16 (1.36) | 3.30 (1.25) | 1.79 (1.39) | 3.10 (1.18) | 2.52 (1.25) | 2.10 (1.48) | 2.39 (1.46) |

Note. Values reported as Mean (*SD*).
