# Supplementary material for: Paths to social licence for tracking-data analytics in university research and services
Source: PLoS One. 2021 May 21;16(5):e0251964. doi: 10.1371/journal.pone.0251964 (PMC8139460; doi:10.1371/journal.pone.0251964)
Supplement: S3 Table — (DOCX) [file pone.0251964.s005.docx]

**S3 Table.** **Parameter posterior distribution summary statistics for model parameters.**

| Parameter | Mode | exp(mode) | 89% HDI | % HDI in ROPE | Rhat |
| --- | --- | --- | --- | --- | --- |
| Intercept | -1.55 | NA | [-2.64, -0.52] | NA | 1.00 |
| Decline Difficulty | -0.05 | 0.95 | [-0.17, 0.04] | 74.3 | 1.00 |
| Private Benefit | -0.04 | 0.96 | [-0.17, 0.06] | 79.44 | 1.00 |
| Participant Benefit | 0.26 | 1.29 | [0.14, 0.42] | 0 | 1.00 |
| Public Benefit | 0.14 | 1.15 | [0.01, 0.29] | 26.93 | 1.00 |
| Disproportionality | -0.09 | 0.93 | [-0.22, 0.04] | 56.02 | 1.00 |
| Sensitivity | -0.24 | 0.78 | [-0.43, -0.10] | 0 | 1.00 |
| Risk of Harm | -0.29 | 0.75 | [-0.45, -0.14] | 0 | 1.00 |
| Trust | 0.49 | 1.63 | [0.34, 0.68] | 0 | 1.00 |
| Data Security | 0.12 | 1.13 | [-0.01, 0.30] | 32.01 | 1.00 |
| Ongoing Control | 0.21 | 1.23 | [0.08, 0.36] | 3.54 | 1.00 |
| Respect for Privacy | 0.54 | 1.72 | [0.36, 0.70] | 0 | 1.00 |
| Scenario random intercept *SD* | 0.42 | NA | [0.11, 0.78] | NA | 1.00 |
| Participant random intercept *SD* | 0.86 | NA | [0.40, 1.28] | NA | 1.01 |
| *Note. ROPE = Region of practical equivalence, HDI = Highest density interval, N = 861.* | | | | | |
