# Supplementary material for: Paths to social licence for tracking-data analytics in university research and services
Source: PLoS One. 2021 May 21;16(5):e0251964. doi: 10.1371/journal.pone.0251964 (PMC8139460; doi:10.1371/journal.pone.0251964)
Supplement: S4 Table — (DOCX) [file pone.0251964.s006.docx]

**S4 Table. Parameter coefficients for predictive model.**

| Parameter | Coefficient |
| --- | --- |
| Intercept | -1.36 |
| Decline Difficulty | -0.15 |
| Private Benefit | -0.12 |
| Participant Benefit | 0.27 |
| Public Benefit | 0.11 |
| Disproportionality | -0.06 |
| Sensitivity | -0.32 |
| Risk of Harm | -0.31 |
| Trust | 0.55 |
| Data Security | 0.32 |
| Ongoing Control | 0.18 |
| Respect for Privacy | 0.55 |
|  | |
