# Supplementary material for: Paths to social licence for tracking-data analytics in university research and services
Source: PLoS One. 2021 May 21;16(5):e0251964. doi: 10.1371/journal.pone.0251964 (PMC8139460; doi:10.1371/journal.pone.0251964)
Supplement: S5 Table — (DOCX) [file pone.0251964.s007.docx]

**S5 Table. Developing final privacy dimensions from workshop dimensions.**

| Dimension from LUMAS workshop | Notes re conversion to final dimensions |
| --- | --- |
|  |  |
| Opt-in/opt-out | *Retained* as ‘Decline difficulty’. |
| Transparency | *Removed* because is difficult to rate from scenario descriptions. |
| Commercial v Non-commercial benefit | *Combined* in final dimensions as ‘Participant Benefit’, ‘Private Benefit’, and ‘Public Benefit’. |
| Personal Benefit |  |
| Who Benefits? How Much? |  |
| Public Benefit v Private Benefit |  |
| Benefit Sharing |  |
| Training and Capacity Building |  |
| Proportionality | *Combined* in final dimensions as ‘Disproportionality’. |
| Scope of Data |  |
| Risk of Harm | *Retained.* |
| Sensitivity | *Retained.* |
| Bona fide | *Combined* in final dimensions as ‘Trust’. |
| Track record |  |
| Conflict of interest |  |
| Evaluation and monitoring |  |
| Equality | *Removed* because is difficult to determine empirically and is hard to define clearly in survey. |
| Security | *Retained* in final dimensions as ‘Data Security’. |
| Use/Reuse/Retention |  |
| Ongoing Subject control | *Retained.* |
| Where/What results released | *Removed* as no variability among scenarios. In each, only aggregate information was to be released, and this is generally the case for university research. |
| Licensing | *Removed* because not used in scenarios, and unlikely to be understood by the general population. |
| Data Linkage | *Subsumed* under ‘Risk of Harm’ dimension. |
|  | ‘Respect for Privacy’ dimension added after literature review. |
